# Deterioration of willow seeds during storage

**DOI:** 10.1038/s41598-018-35476-3

**Published:** 2018-11-21

**Authors:** María Paula López-Fernández, Laura Moyano, María Daniela Correa, Franco Vasile, Hernán Pablo Burrieza, Sara Maldonado

**Affiliations:** 1 Universidad de Buenos Aires, Facultad de Ciencias Exactas y Naturales, Departamento de Biodiversidad y Biología Experimental, Buenos Aires, Argentina; 2 CONICET-Universidad de Buenos Aires. Instituto de Biodiversidad y Biología Experimental (IBBEA)-, Buenos Aires, Argentina; 3 Universidad Nacional del Chaco Austral. Laboratorio de Industrias Alimentarias II. Presidencia Roque Sáenz Peña, Chaco, Argentina

## Abstract

Willow (*Salix* spp.) seeds are able to tolerate desiccation, but differ from typical orthodox seeds in that they lose viability in a few days at room temperature, and in that the chloroplasts in embryo tissues do not dedifferentiate during maturation drying, thus retaining chlorophyll and maintaining intact their thylakoid membranes. In the present study, we investigated the damage generated in willow seeds during storage under appropriate conditions to exclude the eventual generation of reactive oxygen species by photooxidation. To this end, we measured different indicators of molecular damage, such as changes in the fatty acid profile, protein degradation, nuclease activities, and DNA damage, and evaluated normal germination and total germination in seeds stored for one, ten and sixteen years. We found: (i) a decrease in the fraction of unsaturated fatty acids; (ii) changes in the protein profile due to a decrease in protein solubility; (iii) activation of nucleases; and (iv) DNA fragmentation. Taken together, our findings identified programmed cell death as a key mechanism in seed deterioration during storage. We also found that, although the seeds maintained high percentages of total germination, the death program had already started in the seeds stored for ten years and was more advanced in those stored for sixteen years.

## Introduction

Typical orthodox seeds, when stored, age and gradually lose their viability^1–4^. During storage, seeds age mainly by autoxidation^3,5,6^. This autoxidation occurs by peroxidation of polyunsaturated fatty acids, which in turn generate free radicals and reactive oxygen species (ROS). ROS levels initially increase in several diffusion and production centers, thus generating a cascade through specific transduction sensors and inducing the expression of genes related to programmed cell death (PCD). In this respect, Hu *et al*.^7^ detected ROS in elm seeds under controlled deterioration treatment and suggested that PCD plays an important role in seed deterioration.

Modifications in molecules, including fatty acid peroxidation, protein oxidation, nuclease activation, and DNA damage, affect both membranes and organelles^3,5,6,8–12^. Among these modifications, fatty acid peroxidation and glycation of proteins have been reported to be the main biochemical processes involved in the deterioration most frequently associated with seed aging^12–14^.

According to Shaban^12^, autoxidation occurs in two steps: a first step related to the process of aging during the first time of storage, which includes spontaneous oxidation of unsaturated fatty acids and a second step in seeds that have lost their ability to germinate, which affects both saturated and unsaturated fatty acids. Fatty acid oxidation modifies the membrane permeability^15^. Although seeds possess both non-enzymatic and enzymatic defense mechanisms against ROS, according to Bailly and Bailly^16^, the latter are inoperative in a dehydrated state and are only active during seed hydration.

Shaban^12^ asserted that, at moisture contents below 6%, lipid autoxidation may be the main cause of seed deterioration. Above 14% of moisture, water availability increases the activity of hydrolytic oxidative enzymes and lipid peroxidation is promoted. At an intermediate range of moisture (6–14%), lipid peroxidation reaches a minimum because water is enough to serve as a buffer against the free radical attack generated by autoxidation, but not to activate the oxygenases involved in free radical production.

Here, we studied seeds of *Salix nigra* trees, collected every year from 1996 to the present, in order to evaluate the level of autoxidation generated during storage. Mature seeds contain a green embryo covered by a transparent seed coat. Subcellular studies have shown that embryonic cells contain chloroplasts with well-developed grana and reserve proteins and lipids in protein and lipid bodies respectively^17,18^. In addition, cells reserve minerals in the form of phytin and phytoferritin, which are included in protein bodies and chloroplasts, respectively. Unlike typical orthodox seeds, willow seeds tolerate very low water contents, and lose viability in a few weeks at room temperature^18–21^.

We have previously found that seeds of *Salix nigra* exposed to light are very susceptible to photooxidation^19^. In this respect, we proposed that lipid peroxidation is initiated in the granal membranes of the chloroplasts, but then spreads to other cellular membranes^19,20^.

Because in the present study we aimed to exclude the eventual generation of ROS by photooxidation, we investigated the damage generated during storage, by using willow seeds (specifically *Salix nigra* L.) that had been stored for one, ten and sixteen years under the following specific conditions: (i) manipulated in darkness and (ii) stored at −80 °C and 8% water content. We monitored different indicators of molecular damage, including changes in conductivity, changes in the content of photosynthetic pigments, changes in fatty acids, changes in profiles of proteins, activation of nucleases and RNases, and DNA damage, and evaluated total and normal germination.

## Materials and Methods

### Plant material

Seeds of *Salix nigra* L. were collected in mid-November of 2000, 2006 and 2016, from a tree crop of *Salix nigra*, genotype Original (N° 001, Estación Experimental Agropecuaria Delta del Paraná -Instituto Nacional de Tecnología Agropecuaria-INTA). Seeds were manipulated as reported by Maroder *et al*.^21^ and Roqueiro *et al*.^18^, under very low light intensity to reduce the eventual generation of ROS by photooxidation, and stored in sealed containers at −80 °C and around 8% water content expressed in dry weight basis (dwb).

Experiments were repeated with at least three independent biological replicates and, unless otherwise stated, the results were comparable across experiments. Also, due to the small size of the seeds, each replicate consisted of a large number of seeds (approximately 850 seeds contained in 100 mg of seeds).

### Germination tests

Seeds were tested for germination following Maroder *et al*.^21^ and Roqueiro *et al*.^19^. The germination was considered normal when (i) the seedling was erect; (ii) the cotyledons were undamaged and dark green instead of pale green; and (iii) the hypocotyl and the root were well-developed. Seedlings not meeting these criteria were classified as abnormal. Total germination was calculated as the sum of normal germination and abnormal germination. Fresh seeds germinated in 12–24 h (up to 72 h for low vigor seeds). Definitive counting was carried out 6 days after sowing in order to recognize abnormal seedlings with certainty.

### Conductivity tests

Electrolyte leakage from seeds was determined by placing 150 mg of seeds in 6 mL distilled water at 20 °C as previously reported by Roqueiro *et al*.^19^. Conductivity was measured after 3 h of soaking, using an Altronix Model CTX-II conductivimeter (Brooklyn, NY, USA). Results are expressed as µSmg^−1^ of dry seeds (mean of three replicates ± s.d.).

### Determination of pigments

Pigment analysis was carried out as described by Arnon^22^ and Lichtenthaler^23^. The contents of chlorophyll and total carotenes were analyzed simultaneously^24^. To quantify the content of chlorophyll a, chlorophyll b and carotenes, 17 mg (dry weight) of each sample was used; then, 1 mL of N, N-dimethylformamide was added and left in the dark overnight. Then, the samples were centrifuged at 12,000 *g* for 10 min and diluted 5-fold. The optical densities of the supernatant were measured at 664 nm, 647 nm, and 480 nm by means of a spectrophotometer at the same time and pigments were quantified using the equations elaborated by Lichtenthaler^23^.

### Analysis of fatty acids and malondialdehyde (MDA) content

The Bligh and Dyer method^25^ with minor modifications was used to extract the lipid fraction from willow seeds for fatty acid analysis^26^.

Following Burrieza *et al*.^26^, fatty acid composition was determined by gas chromatography after derivation of extracted oils to fatty acid methyl esters (FAME) according to the AOAC Official Method 19^th^ EDITION, 2012. The content of each fatty acid was expressed as mean ± standard deviation.

MDA was determined using the thiobarbituric acid (TBA) reaction (Kai and Feng, 2011). Briefly, seed samples (100 mg) were homogenized in 20% trichloroacetic acid. The homogenate was centrifuged at 18,500 *g* for 20 min and the supernatant was used to evaluate the MDA content. A 0.6-mL sample of 0.5% TBA was added to 0.6 mL of the supernatant, and the mixture was heated at 95 °C for 30 min and then cooled in an ice bath. Then, the mixture was centrifuged at 18,500 *g* for 15 min. The absorbance of the supernatant was measured at wavelengths of 440, 532 and 600 nm, separately.

### Protein analysis

Seeds (50 mg) were ground to a powder with liquid nitrogen. The flour was diluted in 1 mL miliQ water. The homogenate was incubated for 1 h at 4 °C. As described by Burrieza *et al*.^26^, the protein analysis was based on water (Fraction 1) and saline (Fraction 2) solubility. In addition, a final fraction was obtained using a strong denaturing and reducing buffer containing SDS, beta-mercaptoethanol and urea (Fraction 3). The gels were stained with Coomassie brilliant blue R-250. A rabbit polyclonal anti-RuBisCO antiserum raised against RuBisCO’s large subunit (kindly provided by J. J. Guiamet, Universidad Nacional de La Plata, Argentina) was used to carry out a western blot analysis. Western blot analysis was performed following the protocol used by López Fernández *et al*.^27^. Biorad Precision plus (Bio-Rad #161–0373) was used as molecular mass standard. Protein glycation was determined by the dot blot assay, following the protocol described by Wehr and Levine^28^. Briefly, extracts derivatized with 2,4 DNPH were applied to Dry Immobilon-FL PVDF membranes. Derivatized proteins were detected with anti-DNP antibody. Dots corresponding to oxidized proteins were visualized using secondary antibodies conjugated with 3,3′-diaminobenzidine (DAB) from Roche (Merck, Germany) as substrate. For glycation quantitation, a circle to encompass the largest spot on the blot was drawn with the ImageJ software (http://rsbweb.nih.gov/ij/).

### In-gel nuclease activity assay

Nuclease and RNase activities were visualized following SDS-PAGE by in-gel activity assays according to López-Fernández and Maldonado^29^. The samples were frozen in liquid nitrogen before grinding them to a fine powder and homogenized in buffer containing: 10 mM Tris-HCl pH 8.0, 1 mM EDTA, 0.1% (w/v) SDS, 0.1 mM phenylmethylsulphonyl fluoride (PMSF) from Merck KGaA (Darmstadt, Germany), and 1 mM dithiothreitol (DTT). Next, the samples were centrifuged for 15 min at 14,000 g, at 4 °C, and the supernatant was stored and used for the assay. The protein concentrations were determined using a Quick Start Bradford Protein Assay Kit 1 (BioRad #5000201)^30^. Protein extracts from different harvest times were resolved on 12% SDS-PAGE gels containing herring sperm DNA (Biodynamics, Argentina) or torula yeast RNA (Sigma Aldrich, Merck, Darmstadt, Germany). For single-stranded DNase activity assays, DNA was boiled for 5 min prior to pouring in the gels. The samples (40 μg) were incubated for 15 min at 40 °C in buffer (0.125 M Tris pH 6.8, 10% [v/v] glycerol, 2% [w/v] SDS, 0.01% [w/v] bromophenol blue). Following electrophoresis, the gels were washed in a buffer containing 25% 2- propanol and 1 mM EDTA, as previously reported by Leśniewicz *et al*.^31^. Subsequently, the gels containing DNA or RNA were washed twice for 5 min and incubated either overnight (DNA) or for 2 h (RNA), in 25 mM sodium acetate-acetic acid buffer (pH 5.5, containing 0.1 mM ZnSO_4_, 0.2 mM DTT and 1% [v/v] Triton X-100) or 10 mM Tris–HCl neutral buffer (pH 8.0, containing 10 mM CaCl_2_, 0.2 mM DTT and 1% [v/v] Triton X-100) at 37 °C. After incubations, the gels were washed three times for 5 min in buffer (10 mM Tris–HCl pH 8.0, 1 mM EDTA). The gels were stained with 0.01 mg/mL ethidium bromide and photographed as previously described in López-Fernández and Maldonado^29^. All SDS-PAGE results were replicated at least three times.

### DNA isolation and analysis

From each collection genomic DNA was isolated, using the cetyltrimethylammonium bromide (CTAB) method^32^. Seeds were ground in liquid nitrogen into a fine powder and mixed with 400 μL CTAB solution (1.4 M NaCl; 2% [w/v] PVPPM_40.000_; 20 mM EDTA, pH 8.0, 100 mM Tris-HCl, pH 8.0; 2% [w/v] CTAB), and the mix was incubated for 15 min at 70 °C. An equal volume of chloroform:isoamyl alcohol mixture (24:1) was added and, after shaking gently, the mixture was centrifuged for 10 min at 10,000 *g*. The upper aqueous phase was removed, the total DNA was precipitated by addition of 700 μL of 70% [v/v] ethanol, and the DNA was recovered by centrifugation for 2 min at 10,000 *g*. The yield and quality of the DNA obtained were assessed in a Nanodrop 2000 spectrophotometer (Thermo Fisher Scientific). For DNA fragmentation analysis, 20 μg of each sample was separated on a 2% agarose gel and stained with ethidium bromide (final concentration: 0.5 μg/mL). A GeneRuler™ 50-bp Ladder (Fermentas, USA) was used as a reference.

### TUNEL assay

For the TUNEL assay, willow seeds were fixed for 4 h at 4 °C in 4% paraformaldehyde with 0.1 M phosphate buffered saline (PBS) (pH 7.2), dehydrated in a graded ethanol series, and embedded in LRW resin (Polyscience Inc., Warrington, PA, USA) (Harris *et al*., 1995). Semi-thin sections were obtained with an ultramicrotome and mounted on glass slides.

DNA fragments were detected *in situ* using the terminal deoxyribonucleotidyl transferse (TdT)-mediated biotin-16-dUTP nick-end labeling (TUNEL assay), using the “*In situ* Cell Death Detection Kit fluorescein” (Roche, Darmstadt, Germany). TUNEL was conducted according to the manufacturer’s instructions. A negative control was included by omitting the Terminal deoxynucleotide Transferase (TdT) enzyme from the reaction mixture. As a positive control, sections were incubated with DNase I (1 U/µl, Thermo Scientific™) for 15 min before the labeling reaction. Control treatments were prepared for each set of slides. The sections were counterstained with 0.2 µg/mL 4′,6-diamidino-2-phenylindole (DAPI) (Sigma). Images were obtained as previously reported in López-Fernández and Maldonado^29^. The percentage of TUNEL-positive nuclei was calculated from 150 randomly selected nuclei, for each section. At least three whole-mounts of seeds from each harvest were observed.

### Statistical analysis

When applicable, data were analyzed by one-way ANOVA (GraphPad Prism version 6.0), and differences between treatments were determined following Tukey HSD post-hoc test, at *P* ≤ 0.05.

## Results

**Figure 1:**
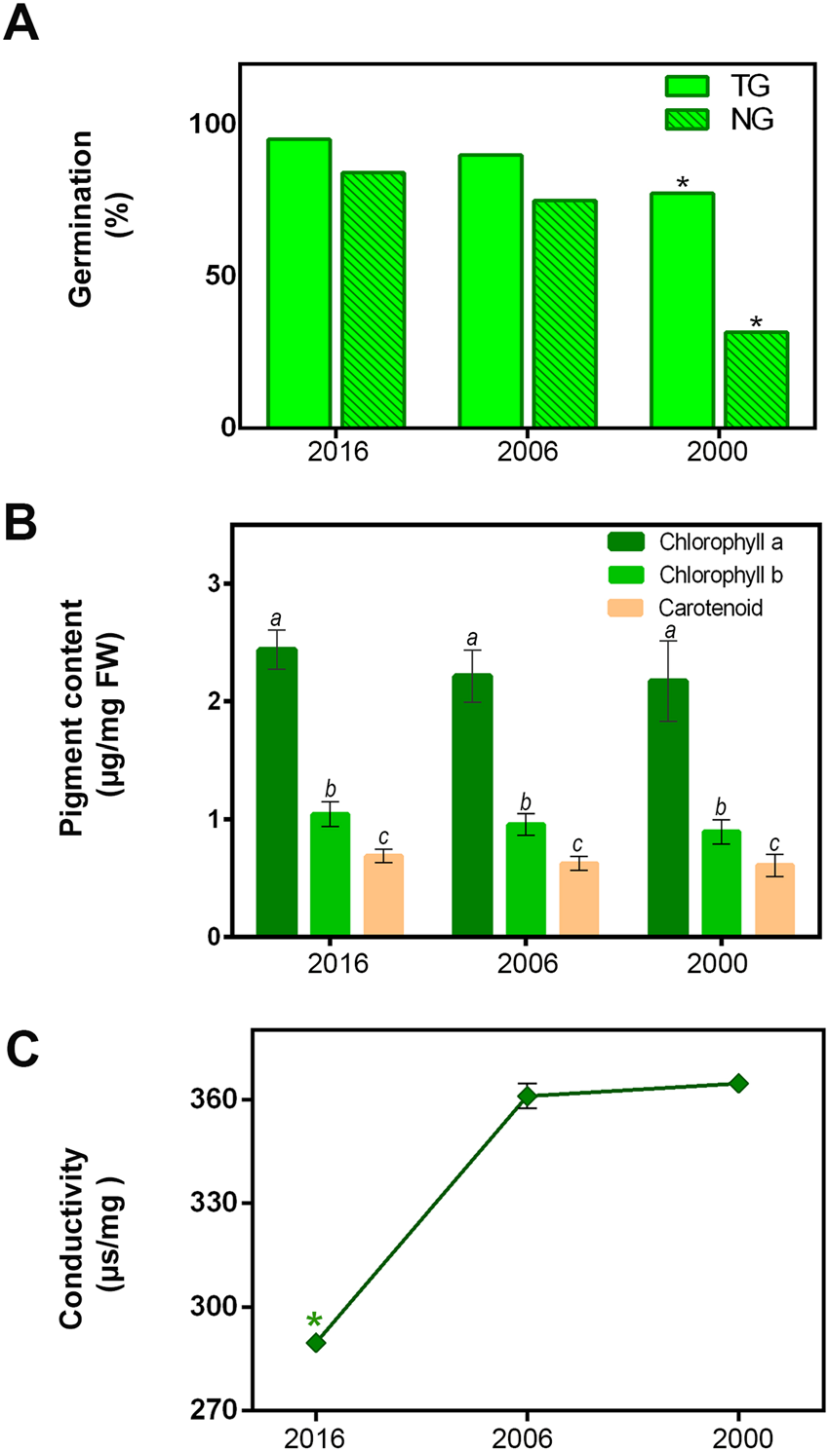
(**A**) Total and Normal and Germination (%) at 6 days after sowing. Both parameters were significantly different only in the oldest collection. Bar values are means of five replicates of 30 seeds ± s.d. Asterisks indicate significant differences according to Tukey’s test (*P* ≤ *0.05)*. (**B**) Contents of chlorophylls and carotenoids during storage. The same letters above columns indicate that contents were not significantly different (*P* ≤ *0.05)* according to Tukey’s test. (**C**) Solute leakage and conductivity. Each point represents the average value of three replicates. The asterisk indicates significant differences according to Tukey’s test (*P* ≤ *0.05*).

Total germination, as evaluated by the standard germination test, varied between 77.5% and 100% (Fig. 1A). In contrast, normal germination decreased progressively in seeds collected in 2016, 2006 and 2000 (84, 75 and 33%^,^ respectively) (Fig. 1A). The damage produced in the seeds was reflected as abnormal and taken into account in the evaluation of normal germination.

A decreasing tendency in the contents of chlorophyll a and b and carotenoids was detected throughout the storage period, but differences were not significant (Fig. 1B).

The electrolyte leakage increased with storage time (Fig. 1C). In addition, permeability was higher in seeds of the older harvests (Fig. 1C) and differences were significant (P ≤ 0.05) between the different harvests.

### Changes in the fatty acid profile and MDA production during storage time

Changes in fatty acid composition provide an indirect measure of the extent of lipid oxidation. Table 1 shows the fatty acid composition and percentage of saturated, monounsaturated and polyunsaturated fatty acids of stored seeds. Samples from different harvests, i.e. stored for different times, presented differences in their fatty acid profile **(**Table 1**)**. Changes in the proportions of saturated fatty acids (SFA) and unsaturated fatty acids were mainly attributed to the oxidative damage occurred during storage. The SFA fraction was slightly affected, which could be explained considering the absence of double bonds in their structure. By contrast, changes in the unsaturated fatty acid fraction were more noticeable. The increase in C18:1 fatty acids of samples stored for a longer time (2006 and 2000 seed collections) was proportional to a very modest decrease in the fractions of C18:2 and C18:3 fatty acids. The oldest samples showed a marked decrease in the relative amount of C18:2 and C18:3 fatty acids, a result in agreement with the more extended damage. The decrease in C18:2 and C18:3 fatty acids was more appreciable in the C18:2 percentages, where the storage time exerted a clear effect. As compared to C18:2, the tendency in C18:3 were less evident.

**Table 1:** Relative percentages of saturated (SFA) and unsaturated and polyunsaturated fatty acids in *Salix nigra* seeds harvested and stored at −80 °C for one, ten and sixteen years.

Fatty acid/(%)	2016	2006	2000
16:0	25.2 ± 0.0^c^	24.9 ± 0.1^b^	24.2 ± 0.0^a^
18:0	3.4 ± 0.0^a^	3.5 ± 0.0^b^	3.4 ± 0.0^a^
18:1	5.6 ± 0.1^a^	11.0 ± 0.0^c^	10.4 ± 0.0^b^
18:2	54.4 ± 0.4^c^	52.2 ± 0.1^b^	51.5 ± 0.0^a^
18:3	10.2 ± 0.2^c^	7.6 ± 0.0^a^	9.4 ± 0.0^b^
20:0	1.0 ± 0.0^a^	1.0 ± 0.3^a^	1.0 ± 0.0^a^
SFA	29.9 ± 0.0^c^	29.2 ± 0.1^b^	28.6 ± 0.0^a^

Mean ±s.d.values followed by different letters within the same row are significantly different according to the ANOVA test at P < 0.05. Tukey’s post-test was used to compare replicate means in each line.

**Figure 2:**
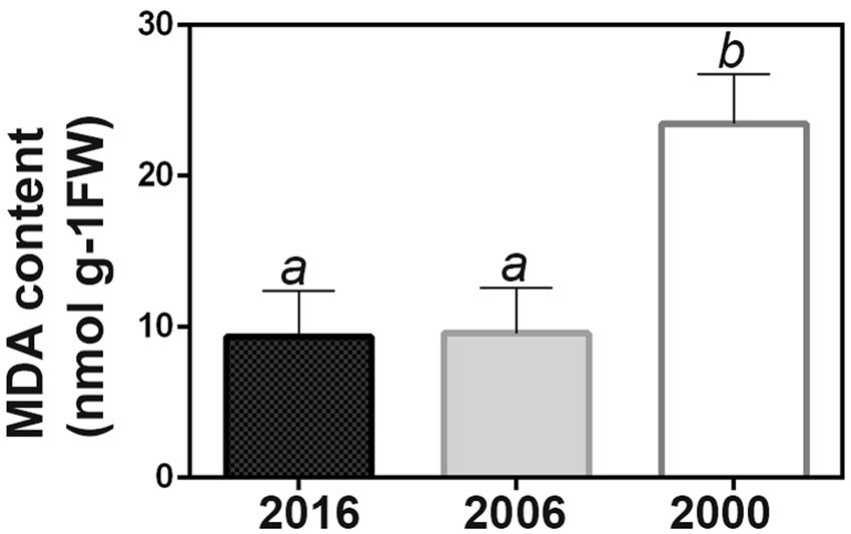
Effect of storage on the MDA content in *Salix nigra* seeds. Bars indicate standard error (n = 3). Mean ± s.d. values followed by different letters are significantly different according to the ANOVA test at P < 0.05. Tukey’s post-test was used to compare replicate means in each line.

MDA, a product of lipid peroxidation, showed a gradual increase during storage time. MDA was detected in the seeds collected in 2016 (Fig. 2) and was progressively higher in the older collections. Differences were significant between successive collections (Fig. 2).

### Protein solubility decreases during storage

**Figure 3:**
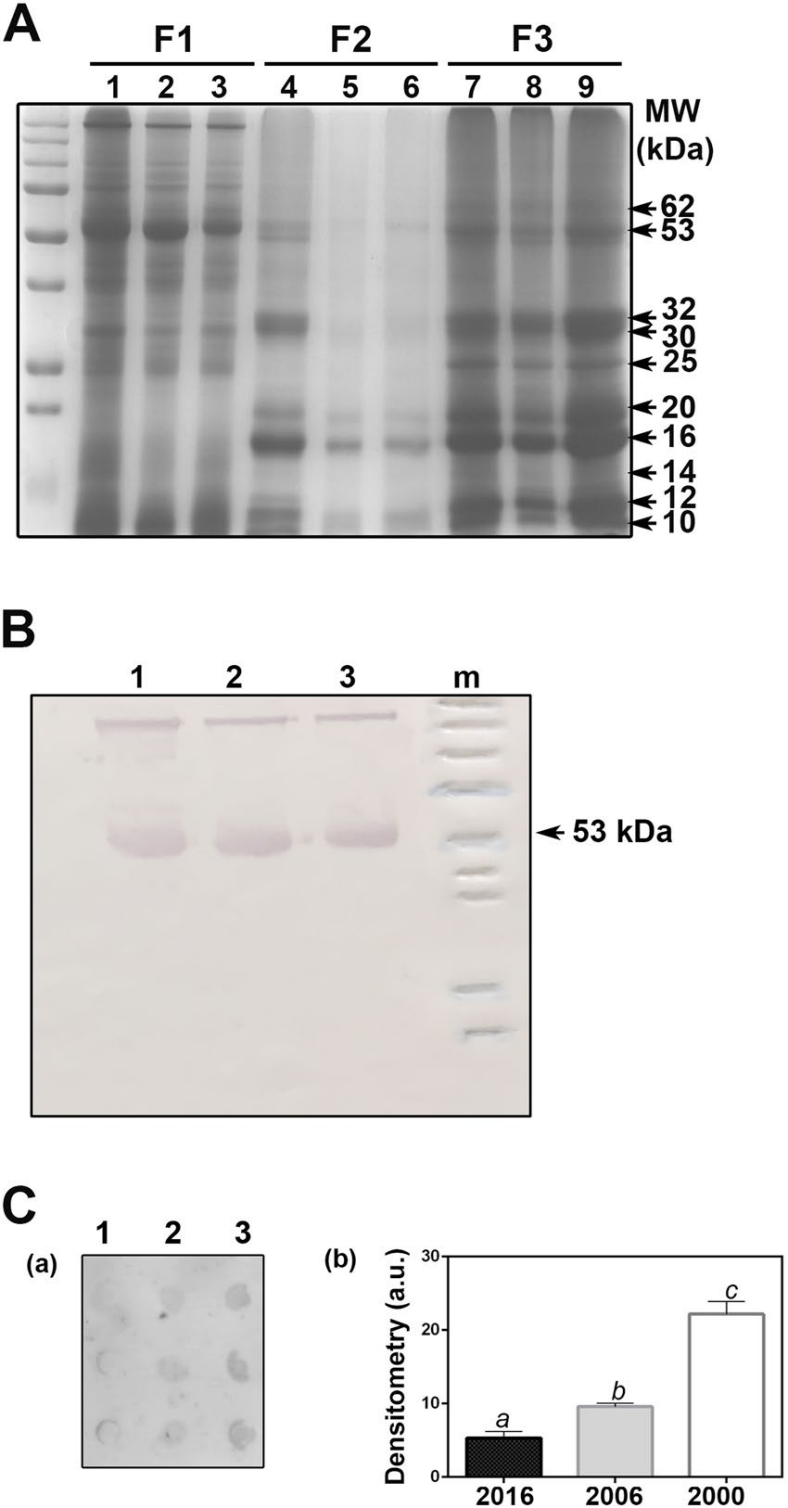
Proteins in *Salix nigra* seeds harvested and stored at −80 °C. (**A**) Protein profile of the three different fractions: water (lanes 1–3), 0.5 M NaCl (lanes 4–6), and denaturing and reducing buffer (lanes 7–9). Lanes 1, 4 and 7 correspond to proteins stored for one year; lanes 2, 5 and 8 correspond to proteins stored for ten years, and lanes 3, 6, and 9 correspond to proteins stored for sixteen years. (**B**) RuBisCo was identified by western blot analysis in fraction 1 (lanes 1–3), using an anti-RuBisCo large subunit global antibody. (**C**) Protein assay of glycation: Lane 1 corresponds to proteins stored for one year; lane 2 corresponds to proteins stored for ten years, and lane 3 corresponds to proteins stored for sixteen years. Each sample was spotted in triplicate. All the rows were loaded with 0.33 µg protein content. Results of the densitometry analysis of dot blot are expressed as arbitrary units (a.u.). Mean ± s.d. values followed by different letters are significantly different according to the ANOVA test at P < 0.05. Tukey’s post-test was used to compare replicate means in each line of two independent experiments.

The different protein fractions were sequentially extracted from the three collections studied (Fig. 3A). The main results were: (i) the proteins resolved into distinct bands that spanned a range of molecular weights from 210 to about 10 kDa; (ii) the band of 53 kDa would correspond to the RuBisCo large subunit^33^. The identity of this band was corroborated by western blot analysis (Fig. 3B); during storage, the large subunit of RuBisCo decreased progressively in fractions 1 and 2 (Fig. 3B); (iii) fraction 2, which should be rich in reserve proteins of the globulin type; here showed a very low number of bands, especially in lanes 5 and 6, which correspond to the seeds collected in 2006 and 2002, respectively (Fig. 3A); (iv) the denaturing and reducing buffer used to obtain fraction 3 showed to have been efficient in the extraction of the remaining proteins, which could correspond to reserve proteins; in this sense, for four species of Salicaceae, i.e. three species of *Populus* (*P. grandidentata, P. balsamifera* and *P. deltoids*) and one of *Salix* (*S. microstachya*), Beardmore *et al*.^34^ reported polypeptides of various sizes (60, 58, 36, 32, 22, 18, and 14 kDa), which would correspond, approximately, to those here observed in fraction 3 (62, 53, 32, 25, 20, 16, and 14 kDa); and (v) as determined by the dot blot method (Fig. 3C) during the storage time, some proteins were modified by non-enzymatic glycation. In fact, glycation in seeds collected in 2000 was significantly different from that in seeds collected in 2006 and 2016, and glycation in seeds collected in 2006 was significantly different from that in seeds collected in 2016 (Fig. 3C; Supplementary Fig. 1).

### Some nucleases and RNases are activated during storage, whereas others decrease

SDS–PAGE was used to identify the activities of the nucleases expressed in the embryo tissues during willow seed storage. These enzymes represent different several classes based on their pH and ion dependence and are associated with nuclear DNA and RNA fragmentation.

**Figure 4:**
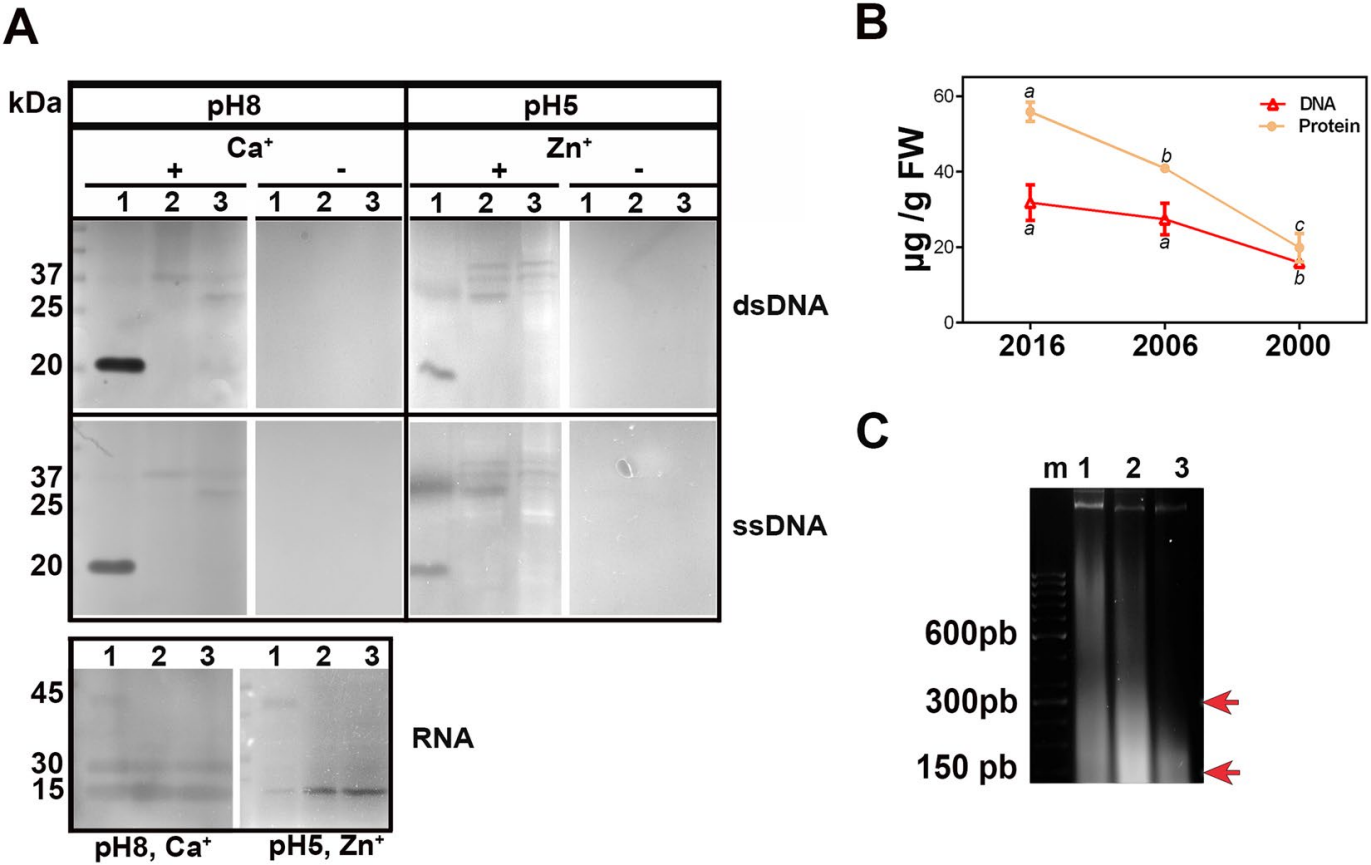
(**A**) Nucleases and RNAses were activated in the presence or absence of Ca^2+^ (left), and of Zn^2+^ (right). Double-stranded (ds) and single-stranded (ss) DNA and RNA were used as substrates. Total proteins were extracted and analyzed at the indicated pH values. (**B**) DNA and protein contents during storage. Numbers 1, 2, and 3 correspond to seeds collected in 2016, 2006 and 2000, respectively. (**C**) Progressive DNA fragmentation can be seen: in the three lanes, one band of high molecular weight allows identifying the non-fragmented DNA; in lanes 2 and 3, DNA smearing and two diffuse bands lower than 600 bp (around 360 and 180 bp) are detected in lanes 2 and 3 (red arrows), m = marker. Mean ± s.d. values followed by different letters are significantly different according to the ANOVA test at P < 0.05 followed by Tukey’s post-test.

When double-stranded DNA (ds DNA) was used as substrate, seeds stored of the three collections showed the following differences: (i) in the presence of Ca^2+^ at pH 8.0, DNase activity gel staining revealed three bands with masses of 20, 30 and 37 kDa; n20 was clearly active in the younger seed collection, and n30 and n37 were active in the oldest one; (ii) in the presence of Zn^2+^ at pH 5.0, DNase activity gel staining revealed four bands corresponding to n20, n30, n37, and n44; (iii) n44 was detected in the older samples, using Zn^2+^ as cofactor; (iv) a similar pattern was detected when single-stranded DNA was used as substrate, although n30 showed a clear preference for single-stranded DNA; (v) no nuclease activity was revealed in the absence of cofactors (Fig. 4A).

When RNA was used as substrate, three bands (n15, n30, and n47) were identified at pH 8 using Ca^2+^ as cofactor. In the presence of Zn^2+^ at pH 5, the n15 band increased its activity in the older samples, suggesting that it is associated with the PCD process. In contrast, the other two bands decreased (Fig. 4A).

Figure 4B shows that proteins decreased during storage and that such decrease was progressive and significantly different between the three collections. The DNA showed no significant differences between seeds collected in 2016 and 2006 but differences were significant between 2006 and 2000 samples.

### DNA electrophoresis revealed that DNA internucleosomal fragmentation increases during storage time

DNA fragmentation is a typical outcome during PCD in many systems. To evaluate DNA integrity, DNA was isolated from seeds of the three collections and analyzed by DNA gel electrophoresis (Fig. 4C). We found that: (i) in seeds collected in 2016 (lane 1), the extracted DNA produced one well-bound band of high molecular weight that allowed identifying the non-fragmented DNA; however a minor smear was detected; (ii) in the DNA extracted from the seeds collected in 2006 and 2000 (lanes 2 and 3, respectively), the band of high molecular weight was clearly weaker; and (iii) a DNA smearing lower than 600 bp was detected in lanes 2 and 3 (red arrows). This clearly demonstrated an advanced point of nuclear DNA degradation, which is associated with the progression of PCD.

### The TUNEL assay detected progressive DNA damage during storage

**Figure 5:**
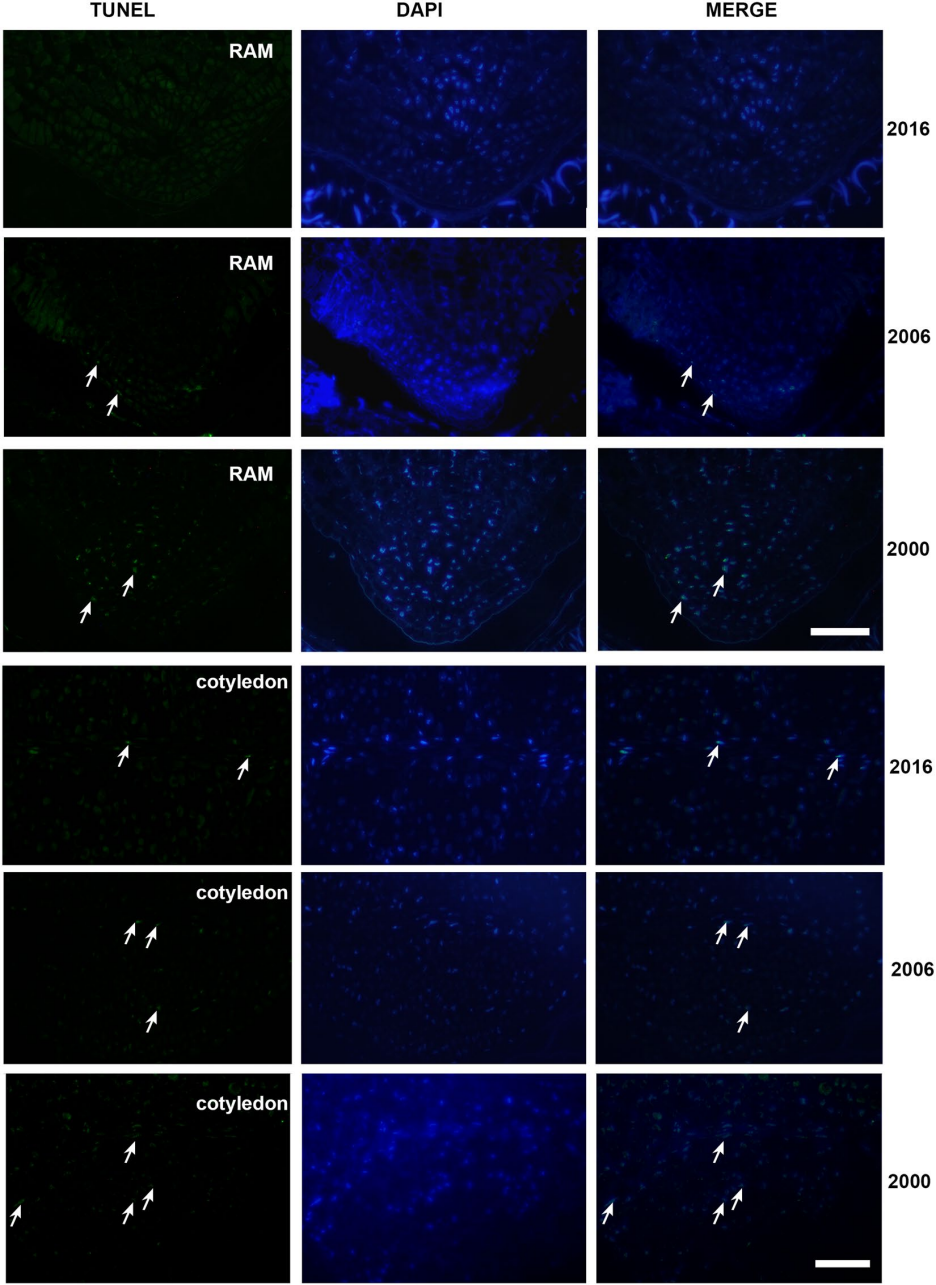
Localization of nuclei with fragmented DNA in *S. nigra* root apical meristem (RAM) and cotyledons from different sample collection as seen following TUNEL and DAPI staining.In each case, the figure is a representative result of the observation of at least three whole-mounts of seeds from each harvest. Scale bar: 50 μm.

**Figure 6:**
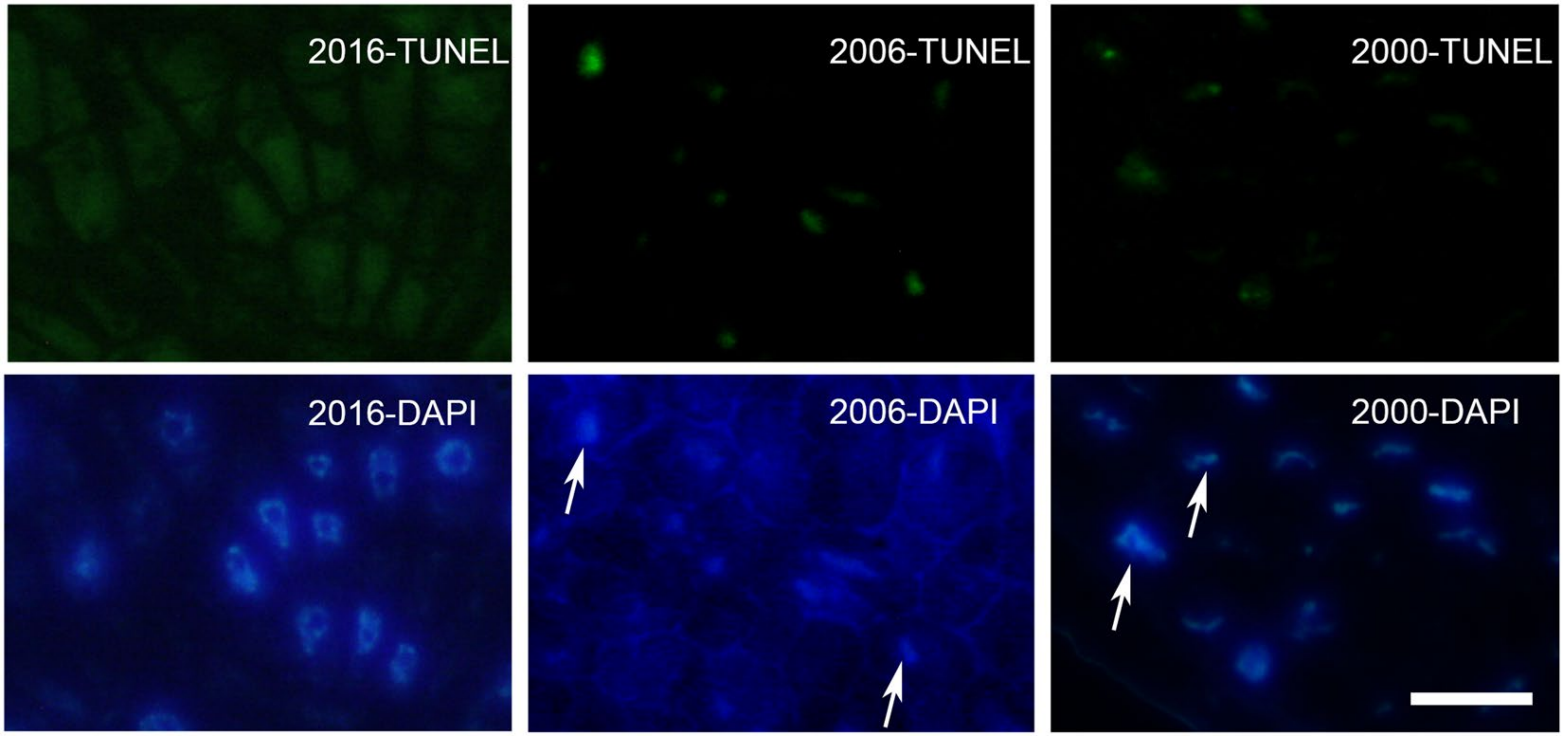
Details of nuclei from the three different harvests following DAPI staining. Scale bar: 12 μm. In each case, the figure is a representative result of the observation of at least three whole-mounts of seeds from each harvest. Scale bar: 12 μm.

Under blue excitation, the TUNEL assay detected nuclear DNA damage as a green fluorescent labeling in the nuclei of cells. Figure 5A shows representative TUNEL assay images of embryo tissues from the three collections. In seeds harvested in 2000, images of the root apical meristem (RAM) exhibited a large number of positively labeled nuclei (Fig. 5). The number of nuclei positively labeled was reduced in seeds stored from 2006 and inexistent in seeds stored from 2016 (except for some nuclei of procambial cells) (Fig. 5). In samples from 2016, only few nuclei of the procambial tissue of the cotyledons presented TUNEL-positive labeling, whereas the rest of the nuclei were TUNEL-negative (Fig. 5). In the cotyledons, the first events of DNA fragmentation were detected in the seeds collected in 2006 and were progressively higher in the older collection (Fig. 5). The percentage of nuclei labeled in the RAM was 0, 7, and 20% in 2016, 2006, 2000 respectively. Analysis of DAPI-stained nuclei by fluorescence microscopy exhibited progressive changes in nuclear morphology (Fig. 6): initially large and round, then nuclei were smaller and became fusiform; also, a progressive increase in chromatin condensation was observed^35–37^.

Positive and negative control treatments were conducted for each set of slides (Supplementary Figs 2 and 3).

## Discussion

Even under optimal storage conditions, seeds tend to lose viability^38^, which has been associated with the accumulation of cellular damage to macromolecules, including lipids, proteins, and DNA^39^. In this study, we monitored different indicators of molecular damage, such as lipid peroxidation, changes in protein profiles, protein glycation, activity of nucleases, and DNA fragmentation, in seeds of *Salix nigra* collected in 2000, 2006 and 2016 and stored according to the protocol previously reported^18–20^, which exclude the eventual generation of ROS by photooxidation. We aimed to evaluate their state of conservation considering that: (i) seeds tolerate desiccation but lose viability in a few weeks at room temperature; and (ii) the chloroplasts of the embryo tissues conserve their chlorophyll and granal membranes^18–21^.

It is extensively agreed that aging in orthodox seeds involves the gradual loss of the integrity of membranes, which are barriers that play an essential role in biochemical or physiological events during germination. The deterioration of membranes involves lipid peroxidation, and this is associated with the leakage of ions, amino acids and sugars^3,11^. In the present study, we found that, although the willow seeds studied maintained very high percentages of total germination, normal germination decreased significantly during storage. Likewise, electrolyte leakage occurred not only in the seeds that had been stored for more than ten years but also in those stored for less than one year, i.e., in a short time following harvest and storage.

It has long been suggested that lipid peroxidation by autoxidation is one of the main causes of seed deterioration. According to Stewart and Bewley^13^, lipid peroxidation and free radical formation are the main causes of the deterioration of oil seeds in storage; during storage, fatty acids are subjected to slow but consistent attack by oxygen, which leads to the formation of hydrogen peroxides, other oxygenated fatty acids and free radicals. The free radicals are unstable and may react and damage nearby molecules. In the absence of enzyme activity, due to the dry state of orthodox seeds during storage, the embryo tissues accumulate oxygenated fatty acids, damaging cellular components and leading to seed deterioration^13^. In this study, we showed that the fatty acid profile of *Salix nigra* seeds was modified during storage. The decrease in the more sensitive unsaturated fatty acid fraction was higher in the samples stored for a longer time, indicating the prevalence of lipid peroxidation at the storage conditions studied.

It worth to note that there was no statistically significant difference in electrolyte leakage of willow seeds collected in both 2006 and 2000, which was unexpected with the results of germination test and MDA measurement. At present, we do not find an explanation to this result. This issue deserves to be deepened.

According to Walters *et al*.^40^, protein oxidation either leads to the formation of smaller molecules with reactive carbonyl or nitrogen groups that easily diffuse through cells or generates an additional reaction between carbohydrates, proteins and nucleic acids, causing intermolecular cross-linking and further degradation into advanced glycation end-products. In *Arabidopsis*, Nguyen *et al*.^14^ confirmed that protein oxidation (carbonylation) is involved in seed deterioration and identified two major storage proteins, crucifernins (12 S globulins) and napins (2 S albumins), which protect seeds from oxidative stress. Here, we analyzed the protein profiles of *Salix nigra* seeds during storage and compared them with those reported in seeds of various species of *Salicaceae* by Beardmore *et al*.^34^. The main and novel differences were: (i) by western blot, we identified that the band of 53 kDa corresponds to the RuBisCo large subunit and that, during storage, this band decreased progressively; (ii) by using saline buffer, we separated a very low amount of polypeptides; and (iii) by using a strong denaturing and reducing buffer, we managed to separate distinct various-sized polypeptides, mostly globulins; such polypeptides were similar in size to those resolved by Beardmore *et al*.^34^ using a saline buffer. We infer that this is caused by a decrease in the solubility of the proteins, which could be attributed to a cross-linking between proteins or between proteins or nucleic acids and carbohydrates; in fact, we found a gradual increase in the content of glycated proteins in the different seed collections, as determined by the dot blot method. This particular issue will be addressed in further studies.

As mentioned above, deterioration in seed quality has been associated with the accumulation of cellular damage to macromolecules, activation of nucleolytic enzymes^41^ and changes in nuclear morphology^35–37^. Progressive deterioration of 18S rRNA has been observed in dry non-viable rye embryos and wheat embryos^42^ and lesions have been found to become amplified with progressive imbibition, either as a result of the intrinsic properties of the rRNA or due to an increase in the activity of other components such as nucleases^43,44^. Progressive rRNA deterioration has also been recorded in embryos of aging seeds of *Triticum durum*^45^. PCD, as revealed by DNA fragmentation and TUNEL assay, has been detected in aged seeds of *Phaseolus vulgaris* and *Helianthus annuus*, coincident with a loss of viability^46,47^. Fragmentation has also been observed to accompany the loss of viability in aged *Secale* cereal seeds^43^. Also, cell death in *Zea mays* (maize) seeds has been associated with a dramatic loss in cellular organization^48,49^. According to Rogers^50^, a significant decrease in DNA content often occurs during plant PCD without forming the characteristic DNA ladder. The occurrence of PCD is more precisely determined when structural analyses are associated with the TUNEL assay^51^. In the present study, DNA degradation, which increased with storage time, was associated with changes in the activity of RNases and different nuclease types (acidic, basic, or salt-stimulated). We inferred that the PCD program was advanced in seeds stored for sixteen years but that the program had already begun in the seeds harvested in 2006. However, no clear internucleosomal DNA fragmentation was observed, but rather a smear indicating DNA degradation, which increased throughout storage time. The lack of detection of a clear laddering could be interpreted as a result of the DNA analyzed from samples composed of approximately 85 seeds with probably different timing of the onset of PCD. We concluded that, during storage, willow seeds deteriorated by autoxidation, and that such deterioration was progressive, affecting all biologically relevant macromolecules, i.e., lipids, proteins and nucleic acids. Taken together, our findings allowed identifying several indicative markers for PCD and suggest that this mechanism is involved in the deterioration that slowly occurs in *Salix nigra* seeds during storage.

## Electronic supplementary material

Supplementary data
