# Supplementary material for: Deterioration of willow seeds during storage
Source: Sci Rep. 2018 Nov 21;8:17207. doi: 10.1038/s41598-018-35476-3 (PMC6249230; doi:10.1038/s41598-018-35476-3)
Supplement: Supplementary file 1 — Supplementary data [file 41598_2018_35476_MOESM1_ESM.docx]

**Deterioration of Willow seeds during storage**

María Paula López-Fernández, Laura Moyano, María Daniela Correa, Franco Emanuel Vasile, Hernán Pablo Burrieza and Sara Maldonado





**Supplementary Figure 1: Protein glycation assay in *S. nigra* seeds harvested and stored at -80°C,** **from different sample collections.** Lanes 1 correspond to proteins stored for one year; lanes 2 correspond to proteins stored for ten years, and lanes 3 correspond to proteins stored for sixteen years. Each sample was spotted in triplicate. All the rows were loaded with 0.33 μg protein content.


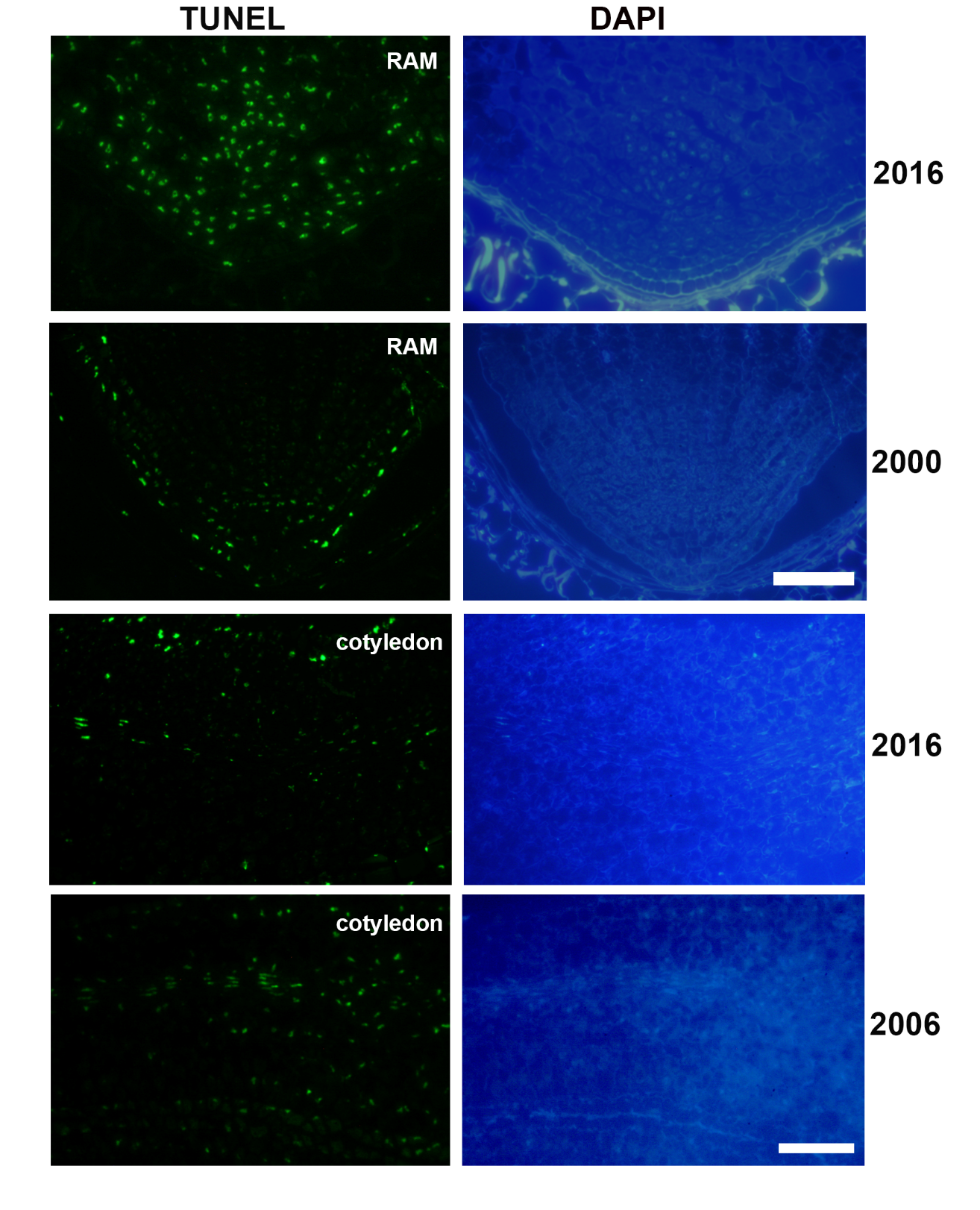


**Supplementary Figure 2: TUNEL-positive control (left column) as compared with DAPI staining (right column) in *S. nigra* root apical meristem (RAM) and cotyledons, from different sample collections.** Sections were permeabilized and then incubated with DNase before assays. In each case the figure is a representative result of the observation of at least three whole-mounts of seeds from each harvest. Scale bar: 50 μm


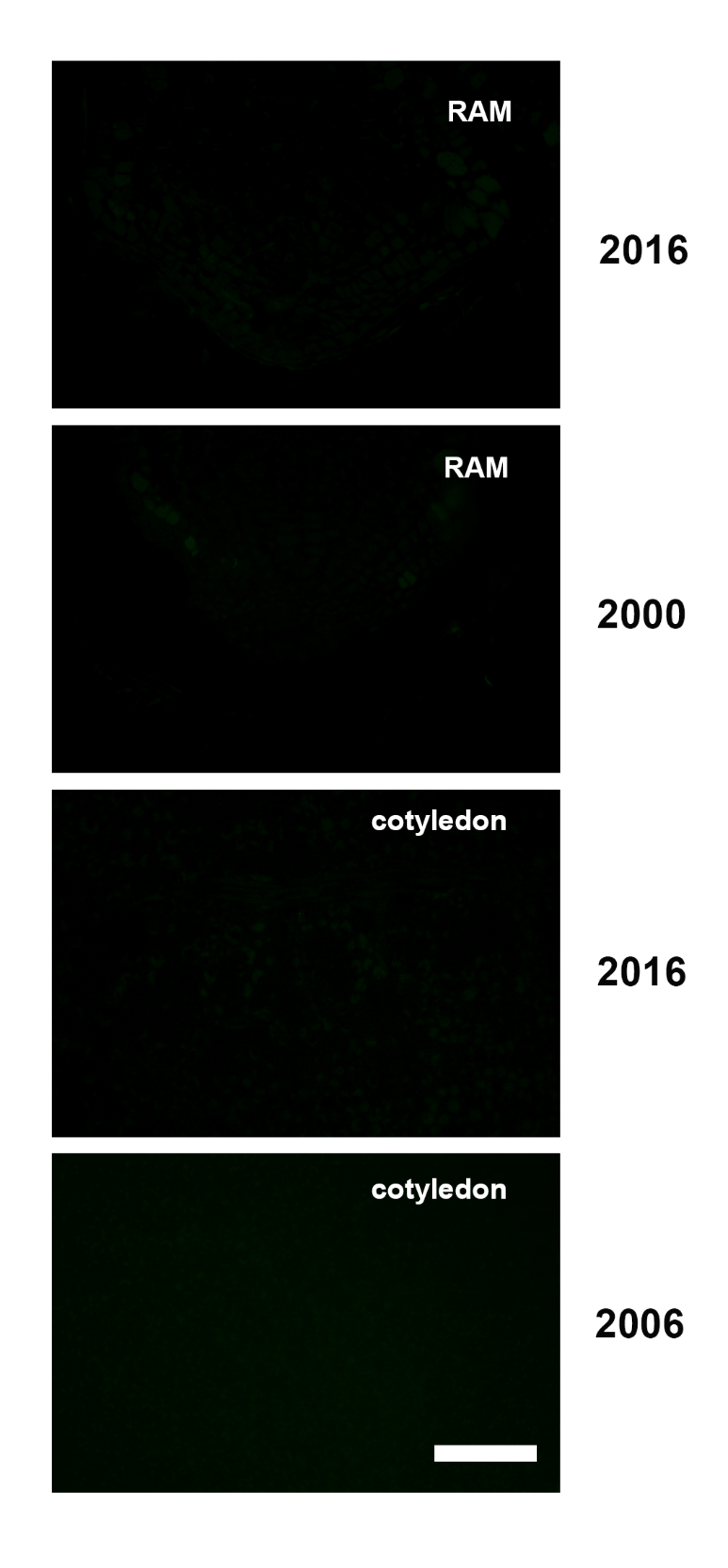


**Supplementary Figure 3: TUNEL-negative control in *S. nigra* root apical meristem (RAM) and cotyledons, from different sample collections.** In each case the figure is a representative result of the observation of at least three whole-mounts of seeds from each harvest. Scale bar: 50 μm
